# The effect of exercise training on endothelial function in postmenopausal women with breast cancer under aromatase inhibitor therapy

**DOI:** 10.1002/cam4.4833

**Published:** 2022-05-18

**Authors:** Barbara Mayr, Bernhard Reich, Richard Greil, Josef Niebauer

**Affiliations:** ^1^ Institute of Sports Medicine, Prevention and Rehabilitation and Research Institute of Molecular Sports Medicine and Rehabilitation Paracelsus Medical University Salzburg Austria; ^2^ Department of Internal Medicine III with Hematology, Medical Oncology, Hemostaseology, Infectious Diseases, Rheumatology, Oncologic Center Paracelsus Medical University Salzburg Salzburg Austria; ^3^ Salzburg Cancer Research Institute with Laboratory of Immunological and Molecular Cancer Research and Center for Clinical Cancer and Immunology Trials Salzburg Austria; ^4^ Cancer Cluster Salzburg Salzburg Austria

**Keywords:** endocrine therapy, high‐intensity interval training, physical exercise capacity, reactive hyperemia index, resistance training

## Abstract

**Background:**

Breast cancer is the leading non‐cardiovascular cause of death in women. In endocrine receptor positive women, aromatase inhibitors (AI) are the therapy of choice despite the fact that a decrease in systemic estrogen levels may result in endothelial dysfunction and eventually in cardiovascular disease. In this study, we assessed whether exercise training (ET), which has repeatedly shown to lead to an improvement of endothelial dysfunction, will also exert this effect in postmenopausal women with AI treated breast cancer.

**Methods:**

Thirty two postmenopausal women with AI treated breast cancer were randomized to an intervention group (ET; 6 months, supervised training plus 6 months without intervention) or control group of usual care (UC; 12 months without intervention plus initial exercise counseling). Endothelial function was assessed via Reactive Hyperemia Index (RHI) measured non‐invasively with the EndoPAT‐System at baseline, 6 and 12 months.

**Results:**

After 6 months of supervised ET, changes in maximal exercise capacity were significantly greater in ET than in UC (∆W: 24.1 ± 11.5 vs. 1.1 ± 8.2 watts; *p* < 0.001). Even though 43.8% of all participants had endothelial dysfunction at baseline, there were no significant group differences in the changes of RHI between ET (∆RHI: −0.1 ± 1.04) and UC (0.02 ± 0.75; *p* = 0.323) after 6 months.

**Conclusion:**

Even though ET led to significantly greater improvement in exercise capacity in postmenopausal women with AI treated breast cancer than exercise counseling only, it did not exert any measurable effects on endothelial dysfunction.

## Graphical abstract

Exercise training led to significantly greater improvement in exercise capacity in postmenopausal women with aromatase inhibitor‐treated breast cancer than exercise counseling only, none the less it did not exert any measurable effects on endothelial dysfunction

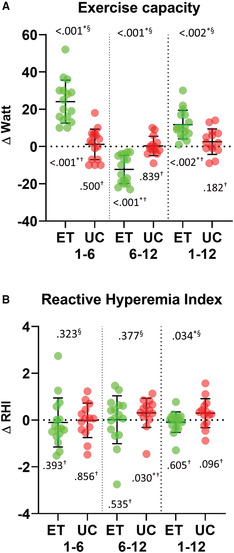

## 1 INTRODUCTION

Breast cancer is the leading non‐cardiovascular cause of death in women and the incidence continues to grow with the aging of the population.^1^ In breast cancer survivors, cardiovascular disease (CVD) is the primary cause of death. These patients are at an even greater risk for CVD‐related mortality compared to women without breast cancer.^2^ This might be possibly due to cardiotoxic side effects of chemotherapy and/or risk factors common to both diseases like increased body mass index or sedentary lifestyle.^2^, ^3^

Estrogens play an essential role in the pathogenesis of breast cancer^4^ and has also an effect on the endothelial function.^5^ In postmenopausal women, estrogen is mainly synthesized in adipocytes by conversion of androstenedione to estrone. Consequently, the majority of studies report that overweight or obesity is associated with increased mortality.^6^ Therefore, controlling body weight and other modifiable cardiovascular risk factors is a priority of long‐term care. The majority of breast cancer patients are endocrine receptor (ER) positive and therefore not only benefit from weight reduction but also from adjuvant endocrine therapy with aromatase inhibitors (AIs).^7^ As a therapeutic result, estrogen levels decrease in these patients, but may increase the risk of endothelial dysfunction, initiation and progression of atherosclerosis, and subsequent cardiovascular events.^8^, ^9^, ^10^ Since exercise training (ET) has repeatedly shown to improve endothelial dysfunction and subsequently reduce the risk of cardiovascular events,^11^, ^12^, ^13^, ^14^ we set out to assess the effects of ET on endothelial function in postmenopausal breast cancer patients treated with AIs.

## 2 MATERIALS AND METHODS

### 2.1 Participants and experimental design

**FIGURE 1:**
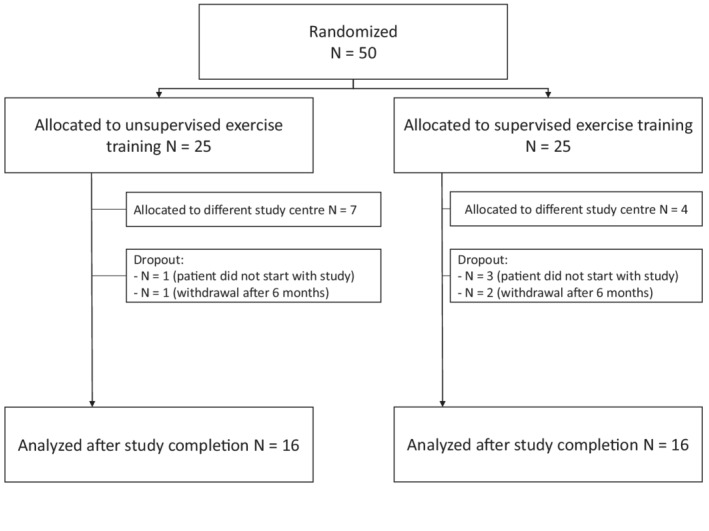
Flow chart of study recruitment

In this randomized controlled trial, we evaluated the effectiveness of 6 months of supervised ET and further 6 months without ET in breast cancer patients on AI therapy.^15^ Women with other relevant chronic diseases or an Eastern Cooperative Oncology Group (ECOG) performance status ≥ III could not participate in this trial. Eligible patients (*n* = 32, 60.9 ± 7.4 years) were randomly assigned 1:1 to ET (ET, *n* = 16) or usual care (UC, (*n* = 16). All subjects underwent counseling on healthy lifestyle changes including nutrition and exercise training. A flow chart of the study recruitment is shown in Figure 1.

Approval from the Ethics committee of the State of Salzburg (415‐E/1290) was obtained before the beginning of the study. All subjects signed written informed consent. The study was registered on ClinicalTrails.gov (NCT01384838). This present work is a sub‐analysis of the “The Working Group on Medical Tumor Therapy” sponsored study previously published elsewhere.^15^

### 2.2 Physical exercise protocols

Patients randomized to ET participated in a supervised, facility‐based training program consisting of high‐intensity interval training (HIIT) and resistance training (RT) for 6 months. In order to meet a minimum of 12 metabolic equivalent of task hours (MET^‐h^) per week of supervised training, exercise volume was set to 2 × 45 min HIIT plus 2 × 30 min of RT per week (see Table 1).^16^ Training sessions were held in the morning, starting with HIIT prior to RT. In addition, subjects assigned to ET were asked to perform an additional hour of self‐reported endurance training per week. Participants randomized to UC got an initial exercise counseling which is part of the usual care. All participants were asked to document their amount and type of physical activity throughout the whole study period in a diary.

**TABLE 1:** Metabolic equivalent of task per training session

Exercise	MET	Time	MET • h^−1^
High‐intensity interval training	7	45 min	5.25
Resistance training	3	30 min	1.5
		Total:	6.75

Abbreviation: MET: metabolic equivalent of task.

### 2.3 Exercise testing

All subjects performed a cycle ergometry (Ergoline GmbH, Bitz, Germany) until exhaustion at baseline, after 6 and after 12 months. The workload started with 20 watts (W) and increased by 10 W every minute until volitional exhaustion. Capillary blood was collected to measure lactate levels before starting the protocol, at 20 W, and every other minute thereafter until maximal exhaustion as well as after 3 and 5 min of recovery. Blood pressure was measured at the same time points, immediately before collecting lactate samples.

### 2.4 High‐intensity interval training

**FIGURE 2:**
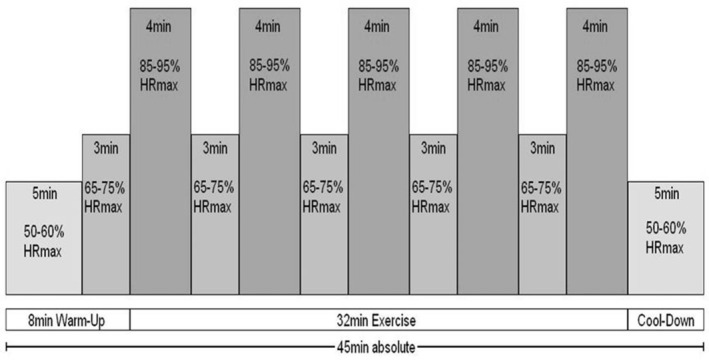
High‐Intensity Interval Training (HIIT) protocol*;* HRmax = peak heart rate

The HIIT‐protocol consisted of 5 × 4min intervals at 85–95% of the individual maximal HR (HR_max_) separated by 3 min of active recovery at 65–75% HR_max_ and a 5‐min warm‐up and cool‐down at 50–60% HR_max_.^17^, ^18^ (Figure 2).

During the first 2 weeks of training, subjects' workload during ergometer training was individually adjusted so that the HR at the end of each interval was 85% of the HR_max_ obtained during baseline exercise testing. Thereafter, workload was increased gradually to a target HR of 90–95% HR_max_. If subjects could not reach 90% HR_max_ then workload was increased gradually during subsequent visits by 2 W per active and recovery interval.

### 2.5 Resistance training

After HIIT, patients performed 30 min of RT: Arm pull‐down, arm dip; butterfly, butterfly reverse; trunk extension, trunk flexion; leg extension, leg curl. Two sets of 12 repetitions were performed during each session at a workload corresponding to 80% of the 10‐repetition maximum, which was tested during the first training session.

### 2.6 Endothelial function studies

Endothelial function was assessed using the EndoPAT 2000 device (Itamar Medical Inc., Caesarea, Israel), which has been validated and used previously to assess endothelial function in other populations.^10^, ^19^, ^20^ Changes of the peripheral arterial tone (PAT) signal to reactive hyperemia (RH) were measured at the fingertip with specially designed finger probes equipped with pressure transducers and an inflating device controlled by a computer algorithm.

The RH procedure consists of a 5 min baseline recording followed by 5 min of blood flow occlusion of the test arm using an upper arm blood pressure cuff inflated above baseline systolic blood pressure. Occlusion of pulsatile arterial flow was confirmed by the reduction of the PAT tracing to zero. After cuff deflation, PAT tracing was recorded for another 10 min. The program automatically normalizes this ratio to the concurrent signal from the contralateral, non‐occluded forearm to correct for confounding variables such as potential systemic effects of unilateral forearm occlusion. Subsequently, this ratio is multiplied with a baseline correction factor to obtain the reactive hyperemia index (RHI). An RHI value of 1.67 was used as a cut‐off value to diagnose endothelial dysfunction.^21^

### 2.7 Statistical analysis

Descriptive statistics are presented as mean ± SD (standard deviation). Statistical significance was assumed at *p* < 0.05. After testing for normality, paired samples were compared using paired t‐test or Wilcoxon‐signed‐rank‐test, while group comparisons were analyzed using Student's *t*‐test or Mann–Whitney *U* test. To see if there were significant differences in the presents of endothelial dysfunction within the study population, a χ^2^‐test was performed.

Pearson's correlations (*r*) were assessed in univariate analyses comparing changes in maximal and submaximal exercise capacity with RHI.

SPSS version 24.0 (SPSS Inc., Chicago, Illinois) was the software used for statistical analyses.

## 3 RESULTS

### 3.1 Subjects characteristics

As already described elsewhere^15^ there were no group differences in time of any baseline clinical characteristics (Table 2). Medication was not altered during the study period. The average time between cancer diagnosis and study inclusion was 22.3 (1–133) months in ET and 29.1 (1–171) months in UC with no significant differences between the two groups. Several women were enrolled after they experienced breast cancer recurrence, sometimes many years after their first diagnosis. The average time between enrollment and AI therapy (14.3 (0–51) months in ET and 15.6 (0–64) months in UC; p = n.s.) or surgical resection (18.1 (1–86) months for ET and 17.3 (1–52) for UC; p = n.s.) was much shorter.

**TABLE 2:** Physical characteristics of the subjects at baseline

Variables		ET (*n* = 16)	UC (*n* = 16)	*p*‐value
Anthropometrics				
Age (years)		61 ± 9	62 ± 9	0.358
Height (cm)		164.4 ± 5.6	164.6 ± 5.5	0.630
BMI (kg/m^2^)		26.6 ± 4.1	26.6 ± 4.4	0.897ǂ
Body weight		71.8 ± 10.4	71.9 ± 11.3	0.785
Body fat (%)		39.4 ± 5.6	38.6 ± 6.8	0.794
Waist circumference (cm)		94.9 ± 11.6	93.8 ± 14.1	0.812
Systolic blood pressure (mmHg)		134 ± 16	136 ± 17	0.580
Diastolic blood pressure (mmHg)		80 ± 9	80 ± 10	0.923ǂ
Performance parameters				
Peak exercise capacity (W)		107 ± 30	115 ± 27	0.410
Peak exercise capacity to weight (W/kg)		1.4 ± 0.4	1.6 ± 0.5	0.365
Peak heart rate (bpm)		147 ± 16	147 ± 19	0.992
Exercise capacity at 2 mmol Lactate/L		55 ± 17	63 ± 27	0.331
Exercise capacity at 4 mmol Lactate/L		83 ± 23	94 ± 29	0.259
Endothelial function				
Reactive Hyperemia Index (RHI)		1.9 ± 0.5	2.0 ± 0.7	0.809ǂ
Cancer characteristics		*N* (%)	*N* (%)	
Her2/neu status	Positive	3 (19)	4 (25)	1.0
	Negative	13 (81)	12 (75)	
Estrogen receptor	Positive	16 (100)	16 (100)	1.0
	Negative	0 (0)	0 (0)	
Progesterone receptor	Positive	13 (81)	0 (0)	0.226 (Fisher's exact test)
	Negative	3 (19)	16 (100)	
Neoadjuvant chemotherapy (+trastuzumab in Her2neu‐positive patients)	Yes	1 (6)	3 (19)	0.600 (Fisher's exact test)
	No	15 (94)	13 (81)	
Adjuvant chemotherapy (+trastuzumab in Her2neu‐positive patients)	Yes	3 (19)	6 (38)	0.433 (Fisher's exact test)
	No	13 (81)	10 (62)	
Recurring cancer	Yes	0 (0)	1 (6)	1.0
	No	16 (100)	15 (94)	

*Note*: Values are presented as arithmetic mean ± SD, *p*‐values are calculated with Student's *t*‐test of if not normal distributed Mann–Whitney *U* test (marked with ǂ).

Abbreviations: ET, Supervised training; UC, Control BMI, body mass index; bpm, beats per minute; W, watt.

### 3.2 Exercise training

**FIGURE 3:**
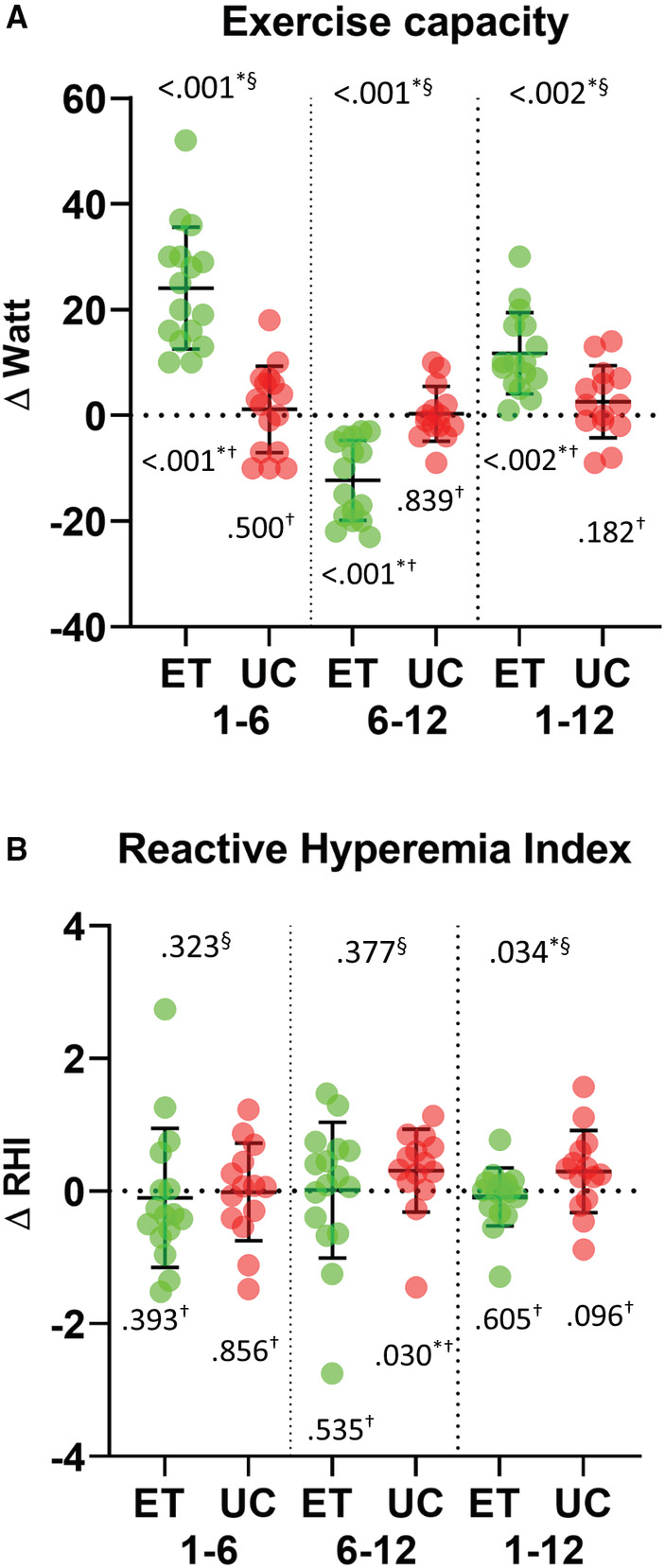
Changes of (A) exercise capacity and (B) Reactive Hyperemia Index (RHI) over the course of the study; Mean values with standard deviation as error bars. Individual values are depicted as dots. ET: supervised training, UC: usual care; green: ET, red: UC; 1–6: supervised intervention phase; 6–12: unsupervised phase; 1–12: whole study period; §: *p*‐values of unpaired *t*‐test respectively Mann–Whitney‐*U*‐test for RHI changes between ET and UC; †: p‐values of paired t‐test within groups respectively Wilcoxon‐test for RHI. N per group = 16, *p < 0.05.

After the intervention phase of 6 months of supervised exercise training the exercise capacity during maximal ergometry increased in ET (107 ± 30 to 131 ± 37 W, *p* < 0.001), whereas no changes occurred in UC (115 ± 27 to 117 ± 27 W, *p* = 0.590). The second half of the study without supervised exercise training resulted in a decrease in the exercise capacity during maximal ergometry in ET (131 ± 37 to 119 ± 34 W, *p* < 0.001), whereas no changes occurred in UC (117 ± 27 to 119 ± 28 W, *p* = 0.839) (Figure 3A). Comparing the changes between both groups, showed significant higher increase in ET (24.1 ± 11.5 W) than UC (1.1 ± 8.2 W; *p* < 0.001). Comparing the changes from 6 to 12 months there was still a significant difference in the changes between groups (ET: −12.3 ± 7.5 W, UC: 0.3 ± 5.2 W; *p* < 0.001).

Furthermore, after 6 months, submaximal exercise capacity increased in ET (2 mmol/L: 55 ± 17 to 77 ± 25 W, *p* = 0.004; 4 mmol/L: 83 ± 23 to 106 ± 27 W, *p* < 0.001) compared to UC (2 mmol/L: 63 ± 27 to 65 ± 28 W, *p* = 0.576; 4 mmol/L: 94 ± 29 to 96 ± 29 W, *p* = 0.668) and no further changes occurred in ET during the second 6 months (2 mmol/L: 77 ± 25 to 73 ± 23 W, *p* = 0.671; 4 mmol/L: 106 ± 27 to 95 ± 26 W, *p* = 0.288), as well as in UC: (2 mmol/L: 65 ± 28 to 59 ± 22 W, *p* = 0.668; 4 mmol/L: 96 ± 29 to 90 ± 26 W, *p* = 0.741).

### 3.3 Endothelial function

The cut‐off value for endothelial dysfunction is commonly set to RHI 1.67,^21^ and 43.8% (ET *n* = 7/ UC *n* = 7) of the included study participants showed RHI values below this threshold (χ^2^‐test *p* = 0.157). After 6 months 50% (ET *n* = 3 / UC *n* = 4) of those improved their RHI values ≥1.67 (χ^2^‐test *p* = 0.593).

After the intervention phase of 6 months of supervised exercise training, there were no changes in the RHI in ET (1.94 ± 0.5 to 1.84 ± 0.8 RHI, *p* = 0.393), as well as in UC (2.04 ± 0.7 to 2.06 ± 0.8 RHI, *p* = 0.856). The second half of the study without supervised exercise training showed no significant changes in ET (1.84 ± 0.8 to 1.85 ± 0.6 RHI, *p* = 0.535), whereas changes occurred in UC (2.06 ± 0.8 to 2.28 ± 0.5 RHI, *p* = 0.030) (Figure 3B). Comparing the changes between both groups, showed no significant differences between both groups (ET: −0.10 ± 1.0 RHI, UC 0.02 ± 0.8 RHI; *p* = 0.323). Comparing the changes from 6 to 12 months there was also no significant difference in the changes between groups (ET: 0.02 ± 1.0 RHI, UC 0.31 ± 0.6 RHI; *p* = 0.377).

To evaluate the relationship between endothelial function and fitness levels we examined Pearsons' correlation between RHI and exercise parameters (exercise capacity at maximum exhaustion, 2 mmol and 4 mmol, peak exercise capacity to weight). There was no correlation between these parameters during baseline examinations or over time (*r* ≤ −0.203; *p* ≥ 0.265).

### 3.4 Self‐reported physical activity diary

The self‐reported physical activity diaries showed that ET performed higher amounts of MET^‐h^ per week in the first 6 months compared to UC (ET: 28.8 ± 8.7 vs. UC: 21.0. ± 13.1; *p* = 0.037), whereas no significant differences were found in the second 6 months (ET: 20.2 ± 8.5 vs. UC: 20.8. ± 11.4; *p* = 0.864). ET reduced their weekly MET^‐h^ in the second period significantly (*p* < 0.001), UC remained unchanged (*p* = 0.632).

## 4 DISCUSSION

The present work is the first study to assess whether exercise training, which has been shown to improve endothelial function in cardiovascular diseases, can counterbalance adverse vascular effects of AI therapy as shown by Maor et al.^8^ Our study's main findings were that supervised high‐intensity interval training and resistance training two times per week for 6 months improved maximal and submaximal exercise capacity, but did not significantly improve endothelial function in postmenopausal women with breast cancer on AI. Blaes and colleagues have previously described that breast cancer patients on aromatase inhibitor therapy have a reduced endothelial function compared to healthy postmenopausal women.^22^ The decline in endothelial function in healthy women occurs in a time‐dependent fashion with an even more pronounced deterioration during menopause. Literature showed that healthy late postmenopausal women (>10 years) have more grave endothelial dysfunction than early postmenopausal women do (<5 years).^23^, ^24^ In keeping with these findings, 43.8% of our patients had endothelial dysfunction at the beginning of the study. In this present study population of postmenopausal breast cancer patients under AI therapy, we could not find a significant or clinically relevant improvement of endothelial function neither in the ET nor in the UC group over time.

Giallauria and colleagues analyzed the effects of exercise training on vascular endothelial function in early‐stage invasive breast cancer patients without AI therapy.^25^ The exercise intervention, as well as the additional dietary intervention were carried out over 1 year. In contrast to our study, Giallauria et al. found significant improvement in the RHI in the exercise intervention group.

Reasons for this discrepancy, besides the AI therapy, which results in reduction of the circulating estrogen concentration by over 90%,^26^ maybe the fact that Giallauria and colleagues enrolled pre‐ whereas we solely enrolled postmenopausal women. As several studies reported that changes in the dietary habits could improve the blood pressure and subsequently the endothelial function,^27^, ^28^ the differences in the results, might as well be also explained by the impact of the dietary intervention. While their intervention lasted 1 year, our study was performed for 6 months of supervised exercise intervention followed by 6 months without supervised exercise routines, a study length, however, long enough to induce changes in endothelial function. Indeed, Lee et al. reported significant improvement in endothelial function measured via brachial artery flow‐mediated dilation in breast cancer patients without AI therapy already after 8 weeks of HIIT, compared to non‐exercising controls.^29^ Taken together, these and our study findings suggest that AI might attenuate the otherwise well described beneficial changes on endothelial function in healthy subjects.

Even though the present study showed no improvement of endothelial function in women on AI therapy, regular physical activity is still warranted also for breast cancer patients on AI therapy. Indeed, we demonstrated that the exercise capacity increased significantly in the intervention group. Physical activity routines to improve exercise capacity are important also for the survival rate of cancer patients,^30^, ^31^, ^32^ as exercise capacity is the strongest prognosticator for cardiovascular as well as all‐cause mortality.^31^, ^33^, ^34^ These results may point at a dissociation of vascular dysfunction as measured by the test system used and the effect of exercise training on cardiovascular morbidity and mortality.

## 5 CONCLUSION

We report for the first time that in postmenopausal women with breast cancer on AI therapy, 6 months of HIIT and resistance training improve maximal and submaximal exercise capacities, without exerting the otherwise well‐known and cardio‐protective effects on endothelial function. Further long‐term studies are needed to not only concentrate on the effects of AI on the morbidity and mortality of postmenopausal women with breast cancer but should also focus on possible detrimental long‐term effects on the cardiovascular system.

### 5.1 Limitation

As this present work is a sub‐analysis of the “The Working Group on Medical Tumor Therapy” sponsored study previously published elsewhere,^15^ we are aware that any assumption that lack of improvement of endothelial function was due to AI therapy, cannot be verified as a control group without AI therapy was missing in this present sub‐analysis. Further, as both study groups were physically active, it is not possible to attribute changes in the endothelial function solely to the presence or absence of physical activity.

## AUTHORS CONTRIBUTIONS

Richard Greil planned, coordinated, and conducted the trial. Barbara Mayr and Josef Niebauer drafted and prepared the manuscript. Barbara Mayr, Bernhard Reich, Josef Niebauer participated in designing and conducting the trial. All authors read and approved the final manuscript.

## CONFLICT OF INTEREST

The authors declare no conflict of interest.

## CLINICAL TRIAL REGISTRATION NUMBER

ClinicalTrails.gov (NCT01384838).

## ETHICAL APPROVAL STATEMENT

Ethics committee of the State of Salzburg (415‐E/1290).

## Data Availability

The data that support the findings of this study are available from the corresponding author upon reasonable request.
